# First Traces and Their Interpretation

**Published:** 1894-06-16

**Authors:** 


### The Study of Man.

### VI.—FIRST TRACES AND THEIR INTERPRETATION.

It is a somewhat puzzling fact that actual remains of early man are peculiarly rare. Hardly any other contemporary species is so poorly represented in our museums, either by whole skeletons, or in particular by skulls sufficiently perfect to support general inferences as to his structure. A main cause of this, no doubt, is the occurrence in prehistoric, as in historic times universally, of the custom of making definite provision for the bodies of the dead. Whether, as among the Indian tribes reported by Herodotus, and more than one more recent example, the corpse is consumed by the survivors, or is exposed to the birds in "Towers of Silence," or buried in earth or rock graves, or burned, wholly or partially, with fire, it almost invariably happens that the remains are put in circumstances least favourable to their preservation during a long period of time. Even those killed in battle, and who might otherwise escape formal burial, are frequently mutilated, or eaten, among barbarous peoples by the tribe which remains in possession of the field. Head-hunting in Borneo is carried on systematically to provide the hunter with slaves in the spirit-world; and there is one instance recorded, where, though the rest of the body is otherwise disposed of, the widow carries her husband's skull by a string round her neck. The best chance of preservation is where the body is embalmed, as in Egypt and the Canary Islands, and committed to a rock tomb or natural cave. Even without embalming bones may be preserved in such rock-shelters for very long periods of time. In Cyprus, for instance, even skulls have been recovered fairly perfect from Bronze Age graves in dry soil and in half-natural limestone caverns; and it is from caves of the latter kind, mostly in the north of Europe, which, of course, has been most thoroughly explored, that the great majority of relics comes of the earliest periods of all.

Similarly in the case of tools and other indications of man's former presence. So long as he confines himself to the use of wooden weapons, or makes his shelters of trees or mud, or small stones, all trace of his work may vanish in a single generation. Even stone implements, which form by far the greater part of the early signs we have of him, are most liable to be worn beyond recognition in the very places where they are most frequent, namely, in and about the valleys of rivers; and, in the earlier stages of workmanship, at all events, are scarcely distinguishable from the gravel in which they occur, even to the eye of an expert. In fact, the existence of such stone tools in Tasmania was not recognised at all until a fortunate observer saw the natives hew down trees and trim them into logs with chips of splintery stone no less rude than those which he had rejected as of natural formation, but which proved, on closer examination, not only to be modelled to give a comfortable grip, but to present actual traces of wear and use.

This Tasmanian example gives us the clue to the method by which such scanty facts as we possess may be construed into a picture of the bodily form and manner of life of primitive man. In remote corners of Continental areas, in the recesses of dense forests, in deserts almost impassable by civilised men, and in particular in oceanic islands, there are to-day, or were at least but yesterday, to be found races of men whose structure and degree of civilisation correspond very closely, in such details as can be compared, with the indications of primitive man and his culture which are to be found in other parts of the world which are now occupied by more developed peoples and civilisations. The Botocados of the Brazilian forests, the Fuegians of extreme South America, the Bushmen of South Africa, the Andaman Islanders, and the aborigines of Australia and of Tasmania—the last, through the inhuman brutality of our colonists, now prematurely extinct—are the best known instances of the class referred to; and the elaborate descriptions which have been published by them within the present century are the best commentary on the now classical accounts of French cave and valley antiquities, and on the increasing collections of our palæethnological museums.

But though these primitive looking peoples present, when compared with the rest of mankind, many features in common, it will be found very hazardous to infer that they are in any sense members of a common group which has thus maintained itself in inaccessible corners of the habitable earth, for both in appearance and still more in their manner of life they will be seen to differ very strongly among themselves. There are probably few in this generation who will care to explain their existence by the crude dogmatic theory of a pre-adamite creation; but there is certainly some danger of a fallacy in the argument that because in certain instances men who are now compelled to inhabit inhospitable regions present a dwarfish appearance, and use rude weapons of stone and bone, they are lineally connected with the makers of similar implements before the rise of the great civilisations, or that the latter were, therefore, necessarily also of ape-like build, or lived in similarly constituted communities. On the morphological question, indeed, a good deal of evidence is already available in opposition to such a view; for while the remains which we have of Neolithic men at all events, by no means bear out the idea of a dwarfish type, the fact that it is only the southern Bushmen, who live in the barren Kalahari desert, and the corresponding desert tribes of Australia, which are reduced in stature and degenerate in features, whereas their kinsmen north of the African desert, and in the more fertile districts of Australia, are by no means deficient in either respect. In the same way there is no clear reason why the small dimensions of the Andamanese, or the extraordinary ugliness—to our eyes—of the Fuegians, should not be largely owing to the miserable conditions of their life, and to the accentuation of much slighter peculiarities by long continued isolation and in-breeding.

What is probably of much more importance is the occurrence in these types of man of features which in greater exaggeration are characteristic of the higher apes: Great length of skull, narrow forehead, and small brain capacity combined with a high degree of prognathism, and of projection of the zygomatic arches; absence, partial or total, of a long or cartilaginous bridge to the nose; great length of arm in comparison with the lower limbs, and of upper arm in comparison with the lower (877—1,000 in Orang, and in Australians, Madurese, or Sudanese, as against an average of about 830 in Germany); loose articulation of the great toe, and ape-like conformation of the "lines" in the skin of the palm. Of course here too the features in question may be of atavistic origin, favoured by peculiar external circumstances; but they are shared in more or less conspicuous degrees by other uncivilised peoples; and present a graduated series which may probably be justly extended to give a valid idea of the early morphology of the species.

In the case of weapons and tools, the argument from analogy is the stronger, in proportion as the problem is simpler, and it is generally safe to infer, for instance, the mode of hafting of a stone axe or arrow head from that still in use here or there for a weapon of the same type. This line of reasoning has, in fact, been strikingly confirmed by the discovery, in Swiss lake-dwellings, and in one or two English sites, of the original handles of neolithic implements, with the blade still in its socket, which confirmed in every detail the conjectural hafting which had been devised for similar specimens.

Hovalacqua. Les Débuts de l'Humanité. Paris, 1882. Tyler's Early History of Mankind.

